# Maximum acceptable communication delay for the realization of telesurgery

**DOI:** 10.1371/journal.pone.0274328

**Published:** 2022-10-06

**Authors:** Akitoshi Nankaku, Masanori Tokunaga, Hiroki Yonezawa, Takahiro Kanno, Kenji Kawashima, Kenichi Hakamada, Satoshi Hirano, Eiji Oki, Masaki Mori, Yusuke Kinugasa

**Affiliations:** 1 Department of Gastrointestinal Surgery, Graduate School of Medicine, Tokyo Medical and Dental University, Tokyo, Japan; 2 Riverfield Corporation, Tokyo, Japan; 3 Department of Information Physics and Computing, the University of Tokyo, Tokyo, Japan; 4 Department of Gastroenterological Surgery, Hirosaki University, Aomori, Japan; 5 Department of Gastroenterological Surgery II, Hokkaido University Faculty of Medicine, Hokkaido, Japan; 6 Department of Gastrointestinal Surgery, Kyushu University, Fukuoka, Japan; 7 Tokai University School of Medicine, Kanagawa, Japan; University of Porto Faculty of Engineering: Universidade do Porto Faculdade de Engenharia, PORTUGAL

## Abstract

**Aim:**

To determine acceptable limits of communication delays in telesurgery, we investigated the impact of communication delays under a dynamic environment using a surgical assist robot. Previous studies have evaluated acceptable delays under static environments. Effects of delays may be enhanced in dynamic environments, but studies have not yet focused on this point.

**Methods:**

Thirty-four subjects with different surgical experience (Group1: no surgical experience; Group2: only laparoscopic surgical experience; Group3: robotic surgery experience) performed 4 tasks under different delays (0, 70, 100, 150, 200, or 300 ms) using a surgical assist robot. Task accomplishment time and total movement distance of forceps were recorded and compared under different communication delays of 0–300 ms. In addition, surgical performance was compared between Group1or Group2 without delay and Group3 with communication delays.

**Results:**

Significant differences in task accomplishment time were found between delays of 0 and 70 ms, but not between delays of 70 and 100 ms. Thereafter, the greater the communication delay, the longer the task accomplishment time. Similar results were obtained in total movement distance of forceps. Comparisons between Group3 with delay and Group1 or Group2 without delay demonstrated that surgical performance in Group3 with delay was superior or equal to that of Group1 or Group2 without delay as long as the delay was 100 ms or less.

**Conclusions:**

Communication delays in telesurgery may be acceptable if 100 ms or less. Experienced surgeons with more than 100 ms of delay could outperform less-experienced surgeons without delay.

## Introduction

A surgical assist robot allows the surgeon to perform operations at a distance from the patient and may make telesurgery possible [1–3]. However, telesurgeries have several challenges to overcome, including communication delays [4, 5]. Theoretically, the greater the communication delay between the transmission of images captured by the camera and the actual movement of the robot in the manipulator, the greater the potential for unexpected intraoperative injuries and fatal complications [4, 6–9].

In September 2001, the world’s first telesurgery on a human was successfully performed. The operation was a laparoscopic cholecystectomy between Strasbourg in France and New York in the United States using a ZEUS^TM^ surgical assist robot and a private line with a stabilized communication delay of around 150 ms [4, 10]. Recent advances in communication technologies have enabled the use of less-expensive public lines with a shorter delay [11–13]. However, the limits of acceptable communication delays during more advanced surgeries such as gastric or colorectal surgeries remain unclear and contentious.

Several studies have been conducted in laboratories to examine the potential impacts of delays in telesurgery. According to Kim et al., a 250-ms delay was acceptable in laparoscopic telesurgery using a surgical assist robot [14]. In addition, Peres et al. and Song et al. stated that telesurgery remained feasible with delays of up to 300 ms and 200 ms, respectively [15, 16]. They also pointed out that the effects of delays varied depending on the difficulty of the operation or task. Moreover, Anvari et al. reported that even a 500-ms delay could be acceptable [17].

These experiments were conducted under stationary environments in which the target object did not move. However, the actual operating field is, to some extent, a dynamic environment, involving factors such as heartbeat, respiratory motions, and peristalsis of the intestines. Studies investigating the effects of communication delays in a dynamic environment are thus warranted.

The present study aimed to evaluate the impact of signal transmission delays on surgery in a dynamic environment and to identify acceptable levels of delay.

## Methods

### Participants in this study

Between October 2020 and February 2021, a total of 35 participants were recruited and categorized into three groups. Group 1 comprised 14 medical students and 6 resident doctors who had never performed any kind of surgery as the primary surgeon. Group 2 comprised 10 young surgeons (mean surgical experience, 5.6 years) who had performed laparoscopic surgery on their own, but had never performed robot-assisted surgeries. Group 3 comprised 5 senior surgeons (mean surgical experience, 17.6 years) who had performed robot-assisted surgeries.

**Fig 1:**
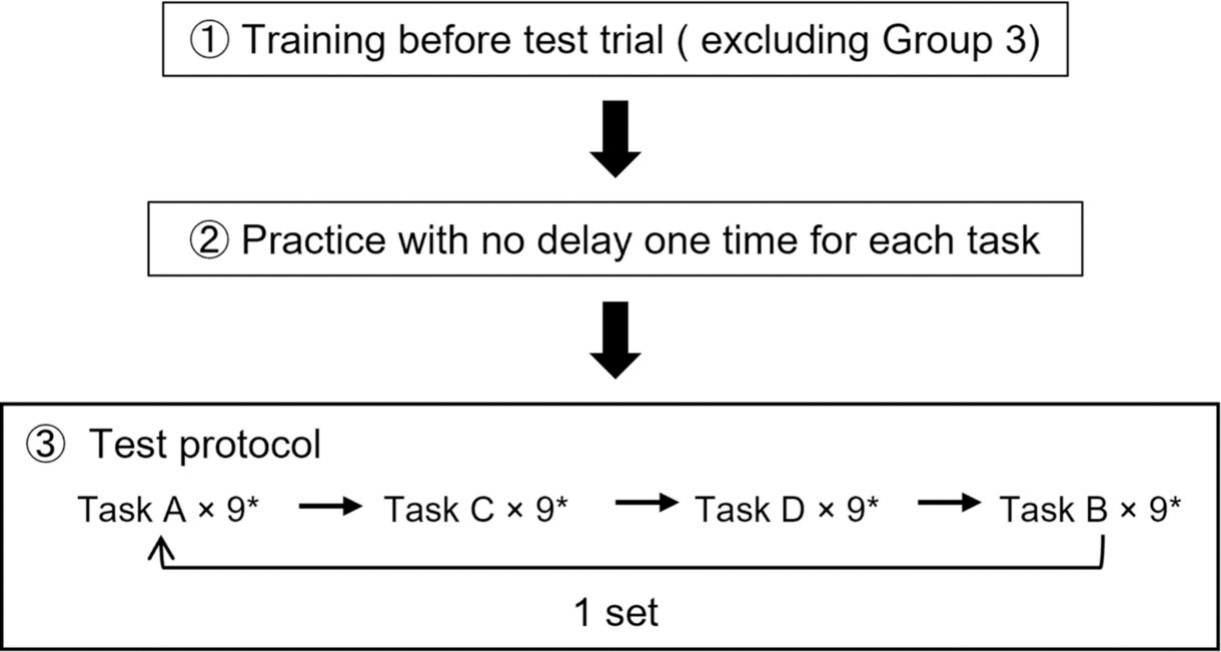
Actual protocol. A total of 4 sets were performed, with the last 2 sets performed on different days. * Each task was performed 9 times with different delay settings, as follows: Delay 0 ms → Delay 0 ms → Delay 0 ms → Delay 70 ms → Delay 100 ms → Delay 150 ms → Delay 200 ms → Delay 300 ms → Delay 0 ms.

Fig 1 shows how the participants performed actual data measurements after training.

### Training protocol for participants in Groups 1 and 2

Participants in Groups 1 and 2 had never performed robot-assisted surgeries, and so complete 2 h of a training program with RobotixMentor (3D SYSTEMS Simbionix, Littleton, CO, USA). During this training, participants were obliged to complete the Robotic Basic skill programs (built into the simulator) at least three times.

### Test protocol

Step 1. All participants were allowed to practice each task only one time, with no delay before the actual trial.

Step 2. Participants performed Task A under no delay 3 times, then with delays in the order of 70, 100, 150, 200 and 300 ms. Finally, the task was performed again with no delay.

Step 3. Step 2 was repeated in the order of Task C, Task D and Task B.

The above cycle (from Step 1 to Step 3) was defined as a one set. All participants performed four sets of tasks in total. The first two sets and the last two sets were performed on different days.

### Tasks (S1 Fig)

#### Task A: Numbering (simple one-handed task)

A board with 3 × 3 holes numbered 1–9 was used. Forceps were inserted into the holes in numerical order. The task began in the neutral position and continued until forceps had been inserted into all holes.

#### Task B: Rope pass (simple two-handed task)

A white rope with red marks at equal intervals was used. The rope was lifted up using right and left forceps, and the surgeon was obliged to grasp only at the marked points. The task was accomplished when the last mark was grasped.

#### Task C: Transfer of sticks (complex task using one hand)

Ten sticks were placed in holes numbered 1–10 on the left side, and five were moved to holes on the right side. Sticks were moved in numerical order. If a stick was dropped, re-grasping was not allowed and the task was continued for the next numbered stick. If the task was completed without dropping any sticks, 5 sticks (in holes 6–10) would remain on the left side. If all sticks were dropped before a total of 5 sticks was moved to the right side, the whole time until the last stick dropped was recorded.

#### Task D: Ring transfer (complex task using both hands)

Six rings placed on rods numbered 1–6 on the left side were moved to rods on the right side in numerical order. Rings on the left side had to be grasped with the left forceps, switched to the right forceps, and placed on the rod with the same number on the right side.

Regarding one-handed tasks, all participants used their right hand. The dominant hand in all but two participants was the right hand, so one-handed tasks were basically performed with the dominant hand.

The fault definitions for each task are described separately below.

### Dynamic target settings

The above tasks were performed on a pedestal that moved horizontally at an amplitude of 1.0 cm at a frequency of 12 times per minute (S2 Fig).

### Experimental system

#### Surgical robot

A prototype of the surgical robot (Riverfield Inc., Tokyo, Japan) was used (S2 Fig). The operation method of the instruments and endoscope is similar to that of the da Vinci surgical system. Motions of the surgeon’s hands are measured by the rotation sensors in the haptic device, and the instrument manipulators reproduce the hand motions of the surgeon. Clutching switches are operated by the middle fingers. We used a 26605AA endoscope (Karl Storz, Tuttlingen, Germany) and CuratOR EX3141-3D display (EIZO, Ishikawa, Japan).

This robot has a start switch, and task completion time was defined as the time from pressing the start switch to press the switch again after finishing the task. Movement distance was automatically measured based on the coordinates of the forceps.

There are no internal electrical or communication latency in the robot system. Mechanical delay is usually less than 100 ms.

#### Network and video configuration

**Fig 2:**
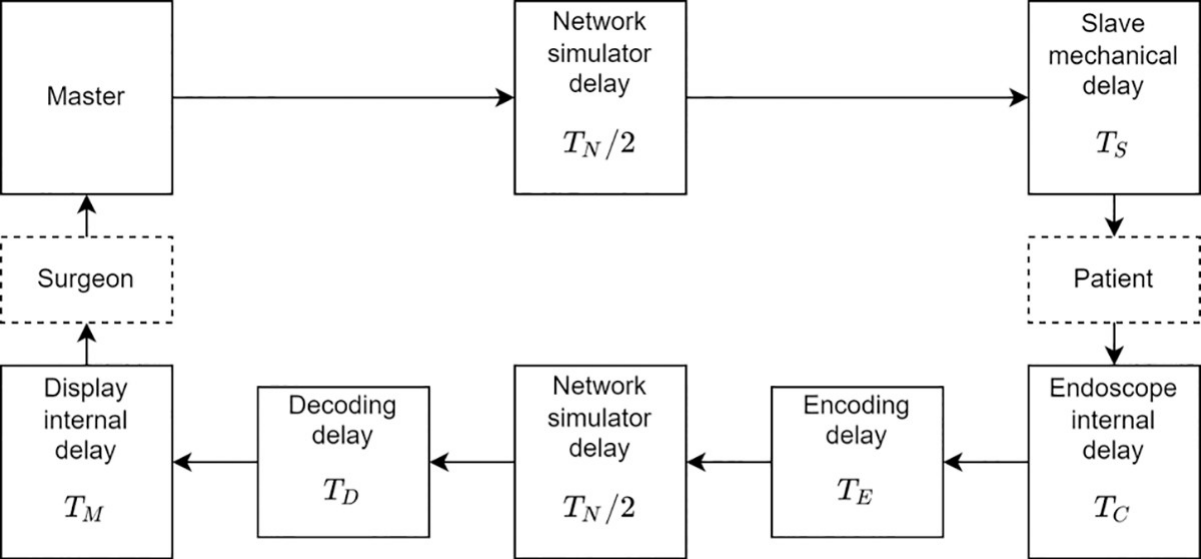
Details of the system used in this experiment. The minimum communication delay was 20 ms, considering the typical performance of domestic communications. The minimum total communication delay was thus 70 ms, including the decoding and encoding times. *T*_*N*_ = {0,20,50,100,150,250}ms, *T*_*E*_+*T*_*D*_≈50ms, Delay defined in this paper *T* is: *T* = *T*_*N*_+*T*_*E*_+*T*_*D*_ = {0,70,100,150,200,300}ms, *Encoder/decoder unused when *T*_*N*_ = 0.

We inserted a network simulator (NetDisturb; ZTI Communications, Lannion, France), which can generate latencies of arbitrary length, between the surgeon console and the patient cart. Fig 2 shows a diagram of the network connection. The surgeon console sends data, and the patient cart controls the positions and orientations of the instruments and endoscope using the received data. The surgeon console sends position and orientation commands 1000 times per second.

Endoscope images are also delayed via the network simulator. We used a video transmitter (Zao-SH; Soliton Systems Inc., Tokyo, Japan), which has a low-latency encoder. Minimum latency from the endoscope to the display is about 40–60 ms, using the transmitter.

#### Latency configuration

We configured the network simulator as shown in Fig 2. Latencies were constant, without jitter or packet losses. Configuration 0 was the control group, in which the latency setting of the network simulator was set to 0 ms and the endoscope image was directly connected to the display without the encoder. Configuration 70 is the typical latency environment for domestic telesurgery, comprising a 20-ms round-trip delay due to network latency and a 50-ms delay from the encoding and decoding processes of the video transmitter. Configuration 300 was the maximum delayed environment in this experiment.

### Delay setting

Our pilot study conducted prior to the present study showed that a surgical tolerance threshold is likely to exist between delays of 100 and 200 ms under dynamic environments. The delays to be generated in the present study were thus 0, 70, 100, 150, 200, and 300 ms.

### Assessment of completion time

Task completion time was defined as the time from when the subject stepped on the start switch to when they completed the task and stepped on the switch again.

### Assessment of movement distance of forceps

Movement was defined as the total distance of both forceps moved from when the subject stepped on the start switch to when they completed the task and stepped on the switch again.

### Number of faults

Task A: Forceps inserted into a hole in the wrong order were treated as a minor fault.

Task B: Grasping an unmarked area was treated as a minor fault, and dropping the rope was treated as a major fault.

Task C: Inserting a stick into the wrong hole was regarded as a minor fault. Dropping a stick was treated as a major fault.

Task D: Dropping a ring or placing a ring on the wrong numbered rod was treated as a minor fault. A ring falling into an area that could not be re-grasped was considered a major fault.

### Statistical analysis

Statistical analysis was performed using R (version 4.0.3) [18]. The Mann-Whitney U test was used to compare continuous values. Two-sided p values were computed, and differences were considered significant for P-values less than 0.05. Task accomplishment time and total movement distance of forceps were compared between delays (0 vs. 70 ms, 70 vs. 100 ms, 100 vs. 150 ms, 150 vs. 200 ms, and 200 vs. 300 ms). As tasks were conducted four times with delay 0 ms, the third measurement was used and all other measurements were omitted for these analyses. We also compared performances of Group 1 or Group2 with no delay (0 ms) and that of Group 3 with delay to assess the impact of surgical experience on performance under the artificially created telesurgery model.

## Results

One subject in Group B enrolled in this study dropped out because he could not complete the practice protocol due to malfunction of the simulator. As a result, a total of 34 subjects participated in this study.

### Completion time

**Fig 3:**
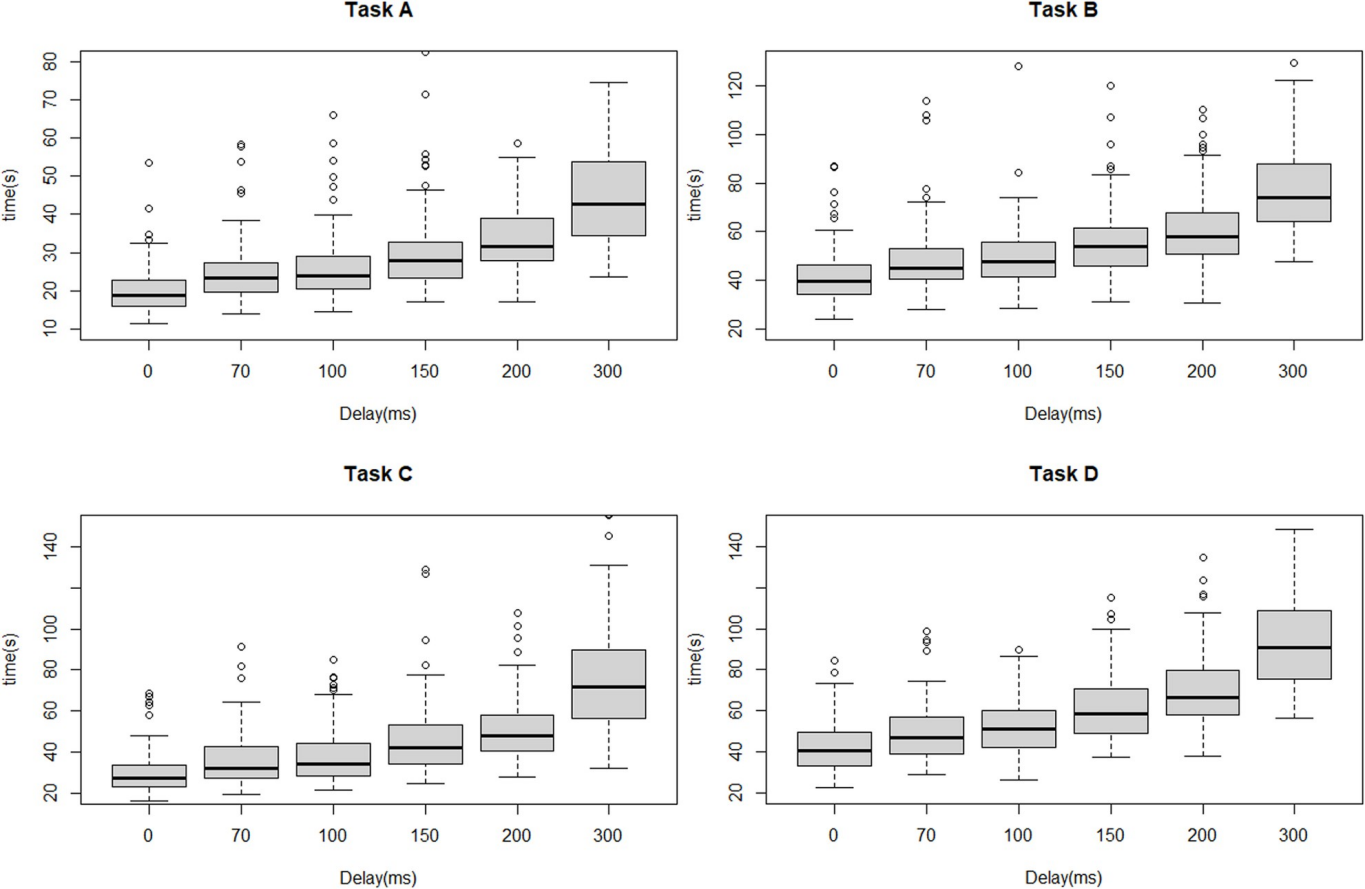
Boxplot of task completion time of each task by all participants.

**Fig 4:**
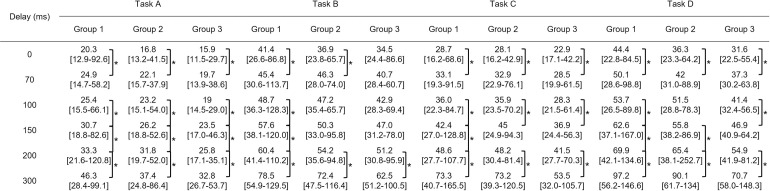
Task completion time for each Group under each delay. * shows significant difference (P value<0.05). Values are median [minimum-maximum].

Fig 3 shows the result of task completion time of all tasks by all participants. In general, task completion time increased between a delay of 0 ms (no delay) and a delay of 70 ms (Task A: 18.8 s vs. 23.3 s, P<0.001; Task B: 39.6 s vs. 44.9 s, P<0.001; Task C: 27.2 s vs. 32.1 s, P<0.001; Task D: 40.7 s vs. 47.0 s, P<0.001), but no significant difference was evident between delays of 70 ms and 100 ms. A significant difference was also found between a delay of 100 ms and a delay of 150 ms (Task A: 23.9 s vs. 27.9 s, P<0.001; Task B: 47.5 s vs. 53.8 s, P<0.001; Task C: 34.1 s vs. 42.3 s, P<0.001; Task D: 51.2 s vs. 58.7 s, P<0.001). Thereafter, the greater the delay, the longer the task accomplishment time, and these differences were significant. A similar trend was found in the subgroup analysis (Fig 4).

### Movement distance of forceps

**Fig 5:**
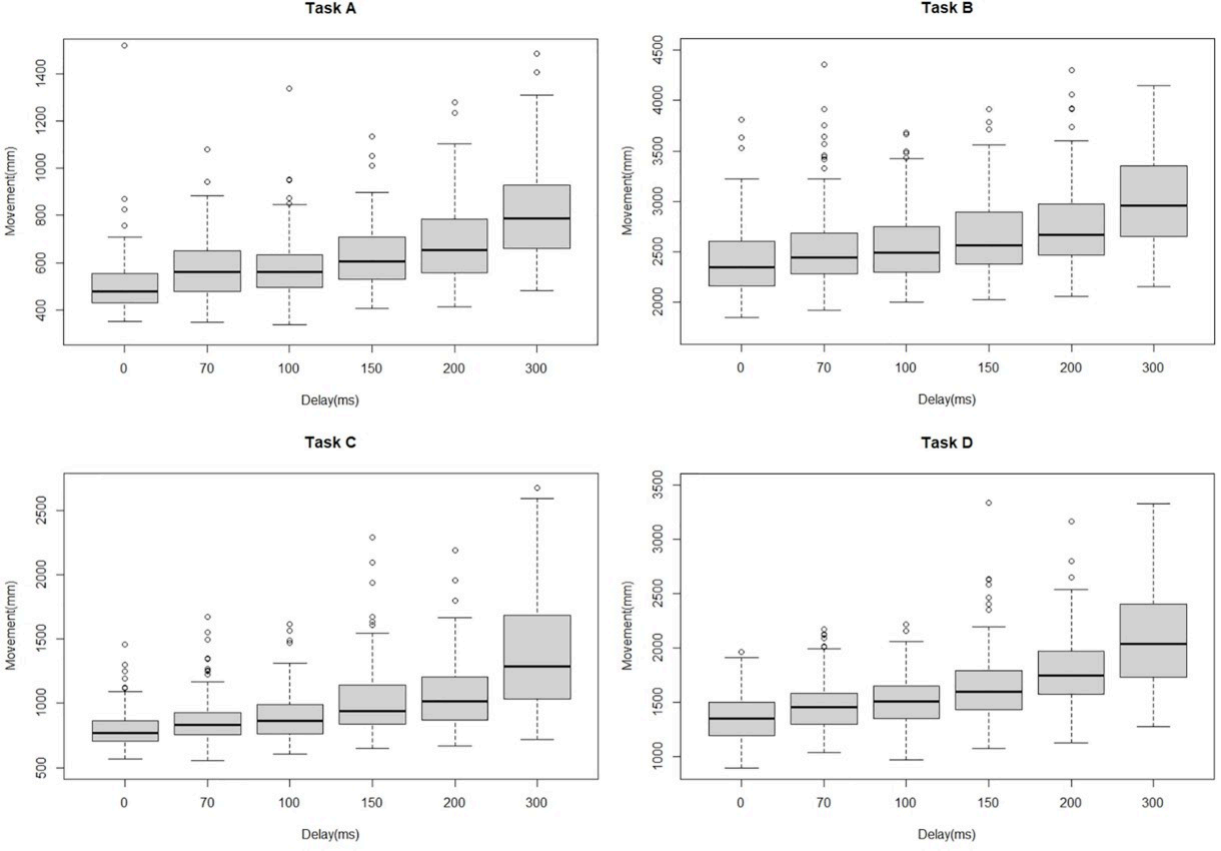
Boxplot of movement distance of forceps of each task by all participants.

**Fig 6:**
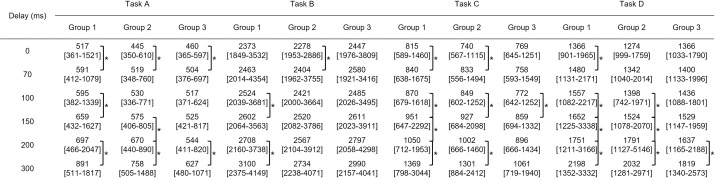
Total movement distance of forceps for each group under each delay. * shows significant difference (P value<0.05). Values are median [minimum-maximum].

Fig 5 shows the result of total movement distance of forceps of all tasks by all participants. Total movement distances of forceps increased with increasing delay. Subgroup analyses revealed similar trends in all groups, although these were less obvious in Groups 2 and 3 (Fig 6). In general, total movement distance extended between a delay of 0 ms (no delay) and a delay of 70 ms (Task A, P<0.001; Task B, P = 0.011; Task C, P<0.001; Task D, P<0.001), but no difference was evident between delays of 70 ms and 100 ms. Thereafter, the greater the delay, the greater the total movement distance. When analyzing the left and right hand separately for the ambidextrous tasks, the performance of the left hand, which is the non-dominant hand for many participants, was slightly more affected by the increased delay. However, these differences did not reach statistical significance.

### Number of faults

With regard to the number of faults, none were seen in Task A. In other tasks, as delay increased, the number of faults generally increased, with a steep rise between delays of 200 ms and 300 ms (S3 Fig). In general, the number of faults did differ markedly under different delays, unlike task completion time or total movement distance. In Task C, no minor faults were observed.

### Comparison between Group 3 with delay and Group 1 or Group2 without delay

**Fig 7:**
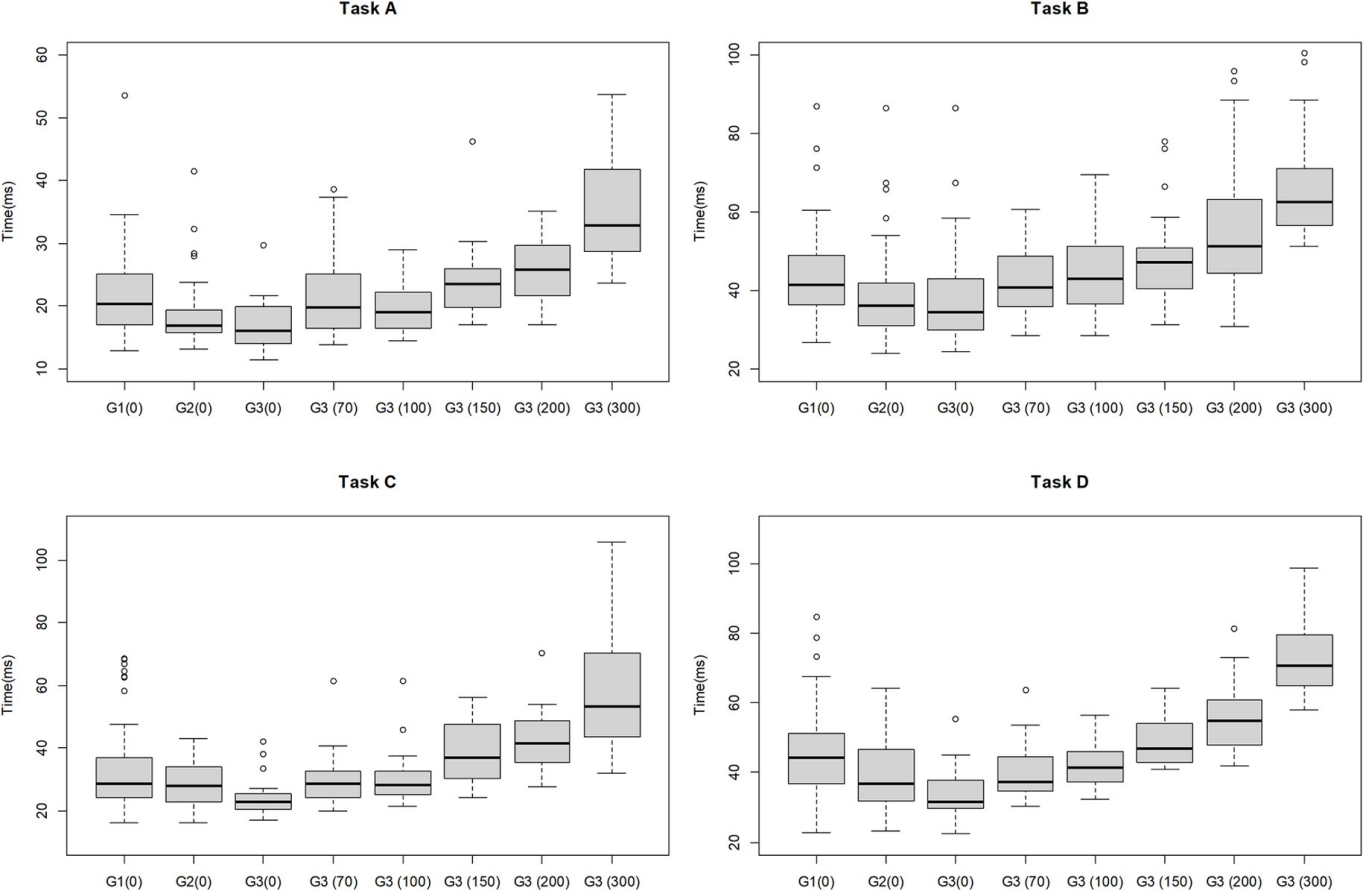
Boxplot of comparing task completion time between Group 3 with delay and Group 1 or Group2 without delay. G1(0), Group 1 without delay; G2(0), Group 2 without delay; G3(0), Group 3 without delay; G3 70, Group 3 with delay of 70 ms; G3 100, Group 3 with delay of 100 ms; G3 150, Group 3 with delay of 150 ms; G3 200, Group 3 with delay of 200 ms; and G3 300, Group 3 with delay of 300 ms.

Fig 7 shows the results of additional analysis in which surgical performance was compared between Group 3 with delay and Group 1 or Group2 without delay. A comparison between Group 3 with a delay of 100 ms and Group 1 without delay showed no significant differences in all tasks for time (Task A: 19.0 s vs. 20.3 s, P = 0.281; Task B: 42.9 s vs. 41.4 s, P = 0.780; Task C: 28.3 s vs. 28.6 s, P = 0.904; Task D: 41.4 s vs. 44.4 s, P = 0.277). Even the performance of Group 3 with a delay of 150 ms did not differ significantly from that of Group 1 without delay. In the comparison between Group 3 with a delay of 100 ms and Group 2 without delay, no significant difference was seen in time (Task A: 19.0 s vs. 16.8 s, P = 0.132; Task B: 42.9 s vs. 36.1 s, P = 0.014; Task C: 28.3 s vs. 27.9 s, P = 0.596; Task D: 41.4 s vs. 36.7 s, P = 0.096). On the other hand, a significant difference was found between Group 3 with a delay of 150 ms and Group 2 without a delay (Task A: 23.5 s vs. 16.8 s, P<0.01; Task B: 47.0 s vs. 36.1 s, P<0.01; Task C: 36.8 s vs. 27.9 s, P<0.01; Task D: 46.9 s vs. 36.7 s, P<0.01). The result of total movement distance of forceps was almost the same (S4 Fig). A comparison between Group 3 with a delay of more than 150 ms and Group 1 or Group 2 without a delay showed significant differences in all tasks except for Task A and Task B between Group 3 and Group 1. The difference in these tasks became significant with delays of 200 ms or more.

## Discussion

The advent of surgical assist robots has furnished surgeons with opportunities to consider telesurgery. Recent technological advances in communications infrastructure, as represented by the development of 5G, may accelerate the clinical use of telesurgery [12]. No technology, however, has yet been developed to completely eliminate communication delays, and a certain amount of communication delay is inevitable. This represents a major issue for surgeons [17], who have to accept some degree of communication delay. According to Marescaux et al., an overall communication delay of up to 330 ms can be acceptable in telesurgery, based on actual remote cholecystectomies safely performed on six pigs and one female human patient, under an average communication delay of 155 ms [4, 5]. No additional studies of acceptable communication delays in telesurgery have been conducted since then under a dynamic environment. In addition, the acceptable minimal delay may well have changed given the innovations in surgical robots over the last 20 years. Several studies have also assessed acceptable communication delays under static environments [14, 16, 17]. However, as target movements make surgical procedures much more difficult, acceptable limits to communication delays seem likely to differ between dynamic and static environments.

To assess the impact of communication delays, four different tasks were tested. Although previous studies have used a variety of tasks with different categories, validated tasks to assess the impact of communication delays on surgical performance have yet to be devised [19, 20]. Since the influence of communication delays under a dynamic environment was considered greater than that under a static environment, the present study adopted four brief tasks: simple single hand; simple both hands; complicated single hand; and complicated both hands. As both hands need to be controlled in actual surgeries, the results of Task D, the complicated both hands task, seem the most important. However, results of other tasks allow a more comprehensive interpretation of the impact of communication delays on surgical performance.

Which surgical outcomes are the most appropriate for assessing surgical performance in artificial dynamic environments remains unclear. In this study, task completion time, total movement distance, and number of faults were evaluated, and the former two were found to be more strongly affected by communication delays than the last one. Longer task completion time and greater total movement distance in accordance with communication delay were expected in dynamic environments, because forceps become difficult to accurately manipulate to a target as communication delays increase, resulting in unintentional back-and-forth movements of the forceps. On the other hand, surgeons might have been able to control the number of faults at the expense of total movement distance of forceps and task completion time. Among these three outcomes, the present study primarily investigated task completion time and used movement distance and number of faults secondarily, but a comprehensive model incorporating all three outcomes may be more useful for evaluating surgical performance and needs to be established and evaluated in the future.

Significant differences in task completion time were generally found between communication delays of 100 ms and 150 ms for all tasks. This result is consistent with what we initially assumed and indicates that the acceptable communication delay is smaller than that reported in previous studies under a static environment. After a delay of 150 ms, we also observed a proportional increase in task completion time as communication delays increased. Performing more complicated tasks, such as clipping and cutting blood vessels as required in actual surgeries, seems difficult under conditions involving a delay of 150 ms or more.

In the present study, we set 70 ms as the minimal delay, as such delay appears unavoidable with the encoding and decoding of information. We found significant differences in performance between delays of 0 ms and 70 ms in most tasks. This result suggests that even very small communication delays may affect surgical quality in dynamic environments. Song et al. [16] reported that surgical quality was unaffected by minimal delay (100 ms), while Rayman et al. concluded even a 300-ms delay did not affect surgical quality under static conditions [19]. The differing outcomes between the present and previous studies are understandable given the substantial differences in environmental conditions. Nevertheless, the findings obtained from the present study indicate that we should think about the impact of communication delay more seriously when actual telesurgery is considered for human patients.

To examine the feasibility of telesurgery, we compared outcomes between Group 3 with delay and Group 1or Group2 without delay. The results of Group 3 with delay of 100 ms were almost the same as those of Group 1 or Group2 with delay of 0 ms. This analysis indicates that the surgical performance of less-experienced surgeons at local sites may be comparable to that of experienced surgeons at remote sites. A certain amount of surgical experience may ensure sufficient quality of surgery under conditions of slight communication delay.

This result shows that telesurgery can already be realized within a relatively small area. Current technologies such as optic fibers can send and receive 4K images under the 100-ms communication delay, including encode and decode times between Japan and East-Asian countries such as China or Korea [21]. Accordingly, telesurgery over a wider area is expected to become possible in the future if communication technologies such as 5G become more widespread.

This study showed some limitations. First, the present study we used a total of 136 sets of data, since each participant (34 doctors) performed 4 sets of tasks. However, this number may have been insufficient to yield robust results. Second, only five doctors included in the present study had experience in performing robotic surgeries. Third, subjects were not blinded to the magnitude of delay, and tasks were always performed in the same order. As familiarity with the delay or task could have affected surgical performance, the order of delay might have affected the results. Therefore, blinding to delay and randomization of task order might be warranted in future work.

In conclusion, communication delays in telesurgery “under dynamic environments” may be acceptable at 100 ms or below. Experienced surgeons with 100 ms of delay could still outperform less-experienced surgeons with no delay. In the near future, experienced surgeons at a distance may be able to advise on or assist in robotic surgeries performed locally by less-experienced surgeons.

## Supporting information

S1 Fig Tasks. Task A: Numbering (Simple One-Handed Task), Task B: Rope Pass (Simple Two-Handed Task), Task C: Transfer of Sticks (Complex Task Using One Hand), Task D: Ring Transfer (Complex Task Using Both Hands).
(TIF)

S2 Fig Equipment used in the experiment. (A) Dynamic Target Setting repeating 12 horizontal movements per minute. (B): A prototype of the surgical robot (Riverfield Inc.).
(TIF)

S3 Fig Total number of faults of each task. No one made any faults in Task A. In Task C, no minor faults were observed.
(TIF)

S4 Fig Boxplot comparing movement distance of forceps between Group 3 with delay and Group 1 or Group 2 without delay. G1(0), Group 1 without delay; G2(0), Group 2 without delay; G3(0), Group 3 without delay; G3 70, Group 3 with delay of 70 ms; G3 100, Group 3 with delay of 100 ms; G3 150, Group 3 with delay of 150 ms; G3 200, Group 3 with delay of 200 ms; G3 300, Group 3 with delay of 300 ms.
(TIF)

S1 Dataset (XLSX)
