# Supplementary figures and images for: Maximum acceptable communication delay for the realization of telesurgery
Source: PLoS One. 2022 Oct 6;17(10):e0274328. doi: 10.1371/journal.pone.0274328 (PMC9536636; doi:10.1371/journal.pone.0274328)

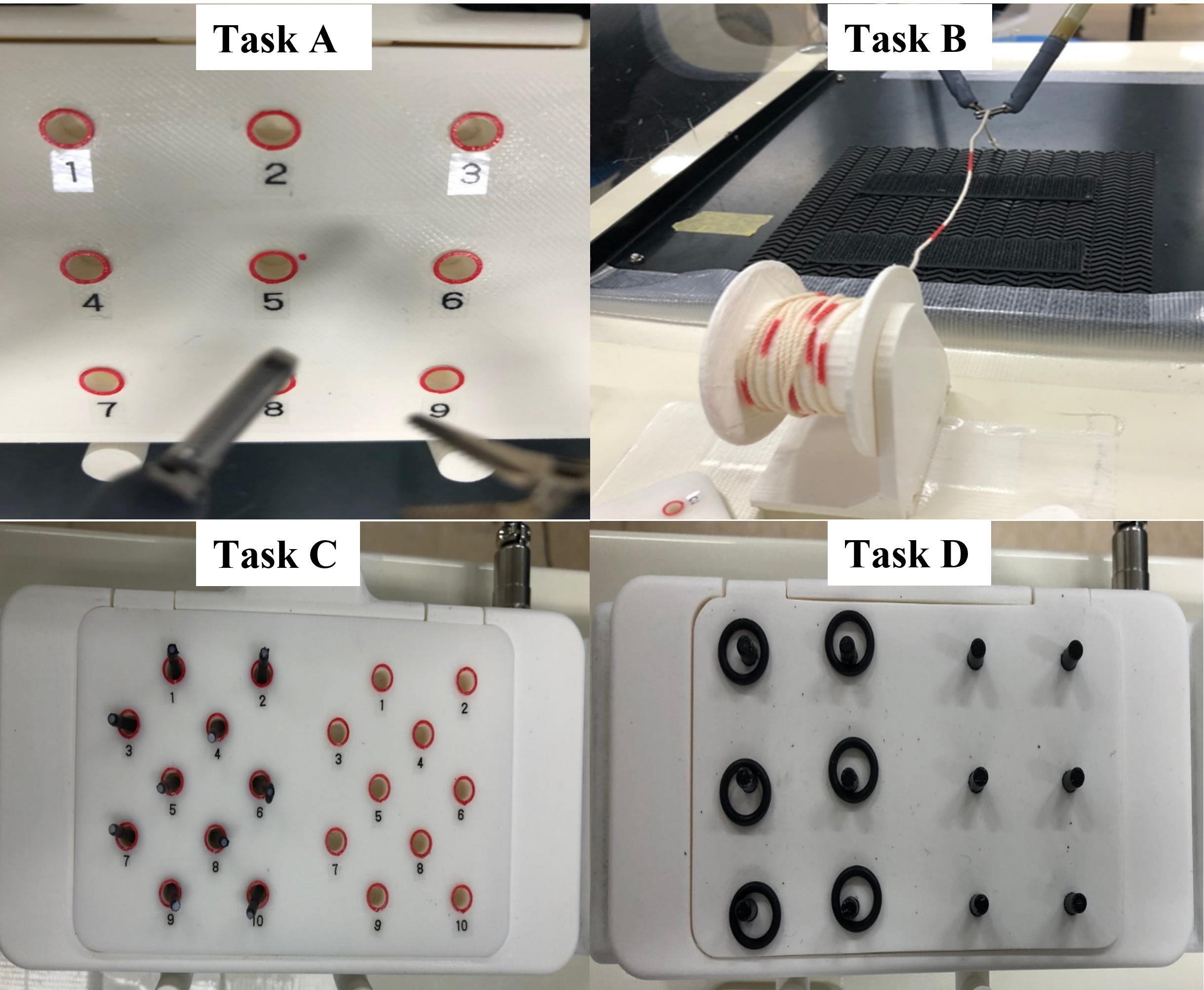

Supplement: S1 Fig — Task A: Numbering (Simple One-Handed Task), Task B: Rope Pass (Simple Two-Handed Task), Task C: Transfer of Sticks (Complex Task Using One Hand), Task D: Ring Transfer (Complex Task Using Both Hands). (TIF) [file pone.0274328.s001.tif]

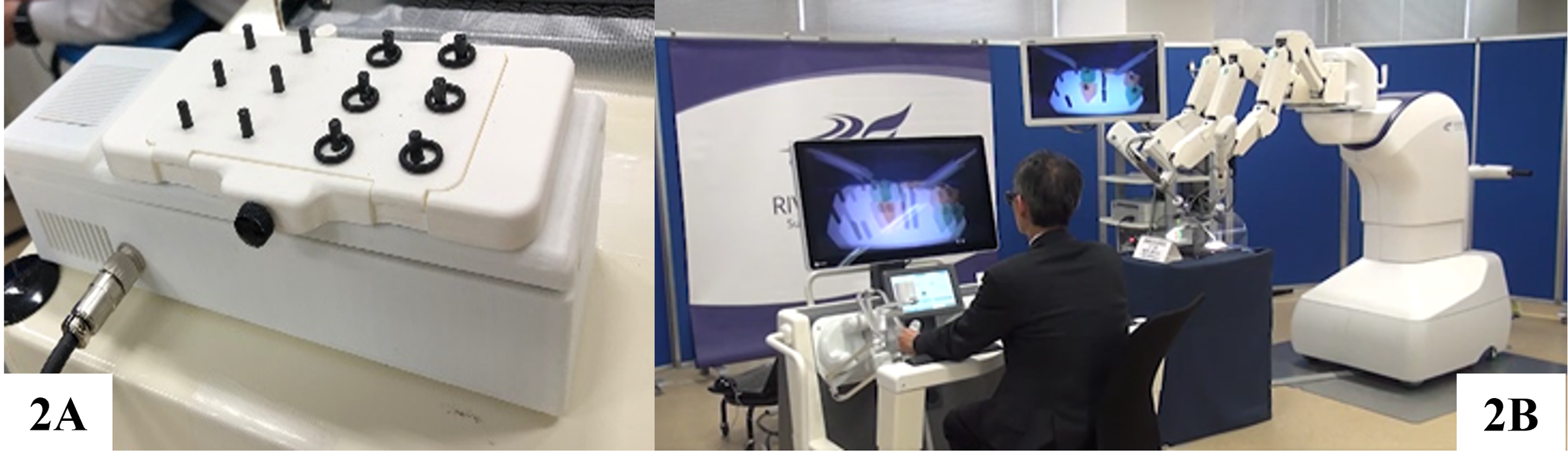

Supplement: S2 Fig — (A) Dynamic Target Setting repeating 12 horizontal movements per minute. (B): A prototype of the surgical robot (Riverfield Inc.). (TIF) [file pone.0274328.s002.tif]

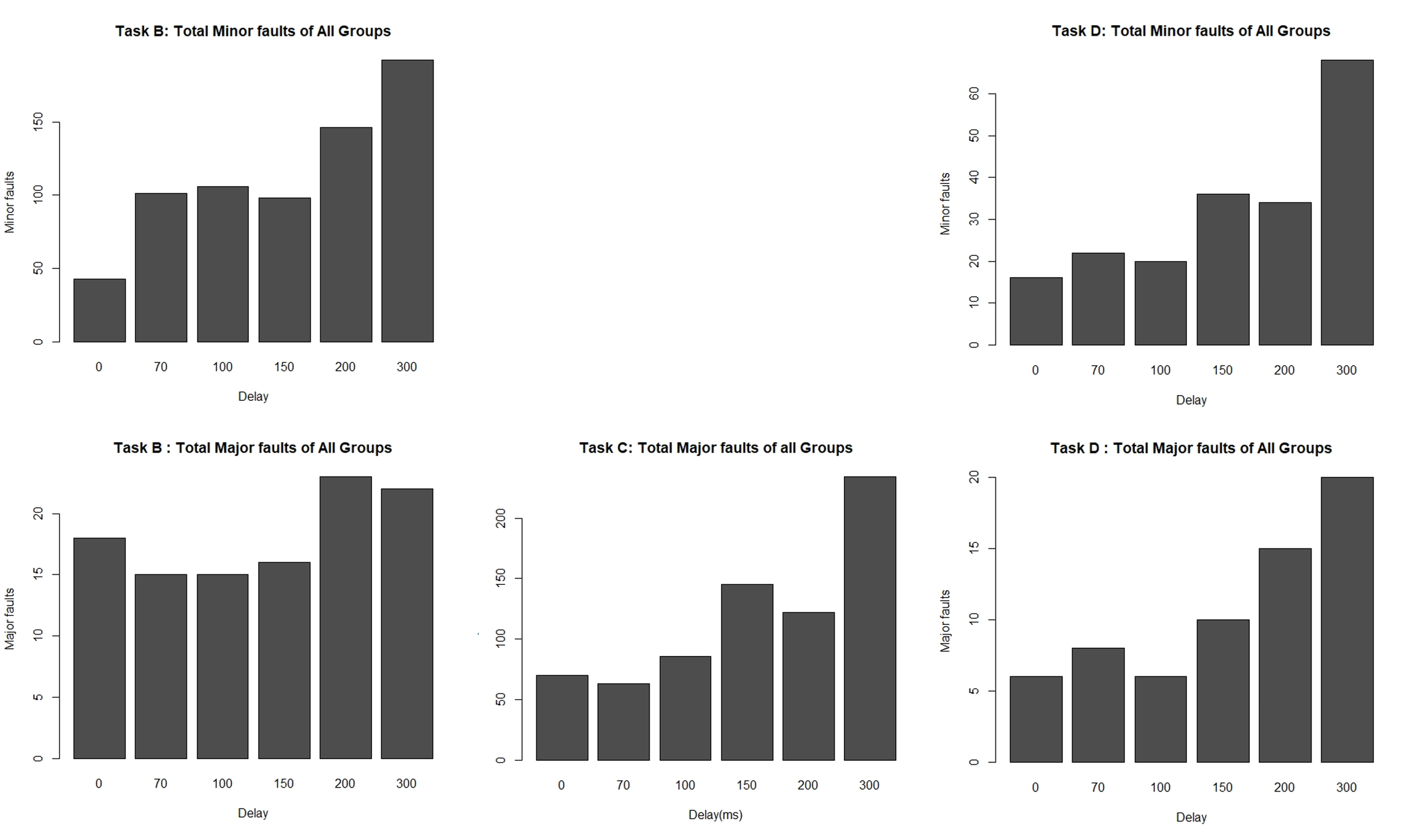

Supplement: S3 Fig — No one made any faults in Task A. In Task C, no minor faults were observed. (TIF) [file pone.0274328.s003.tif]

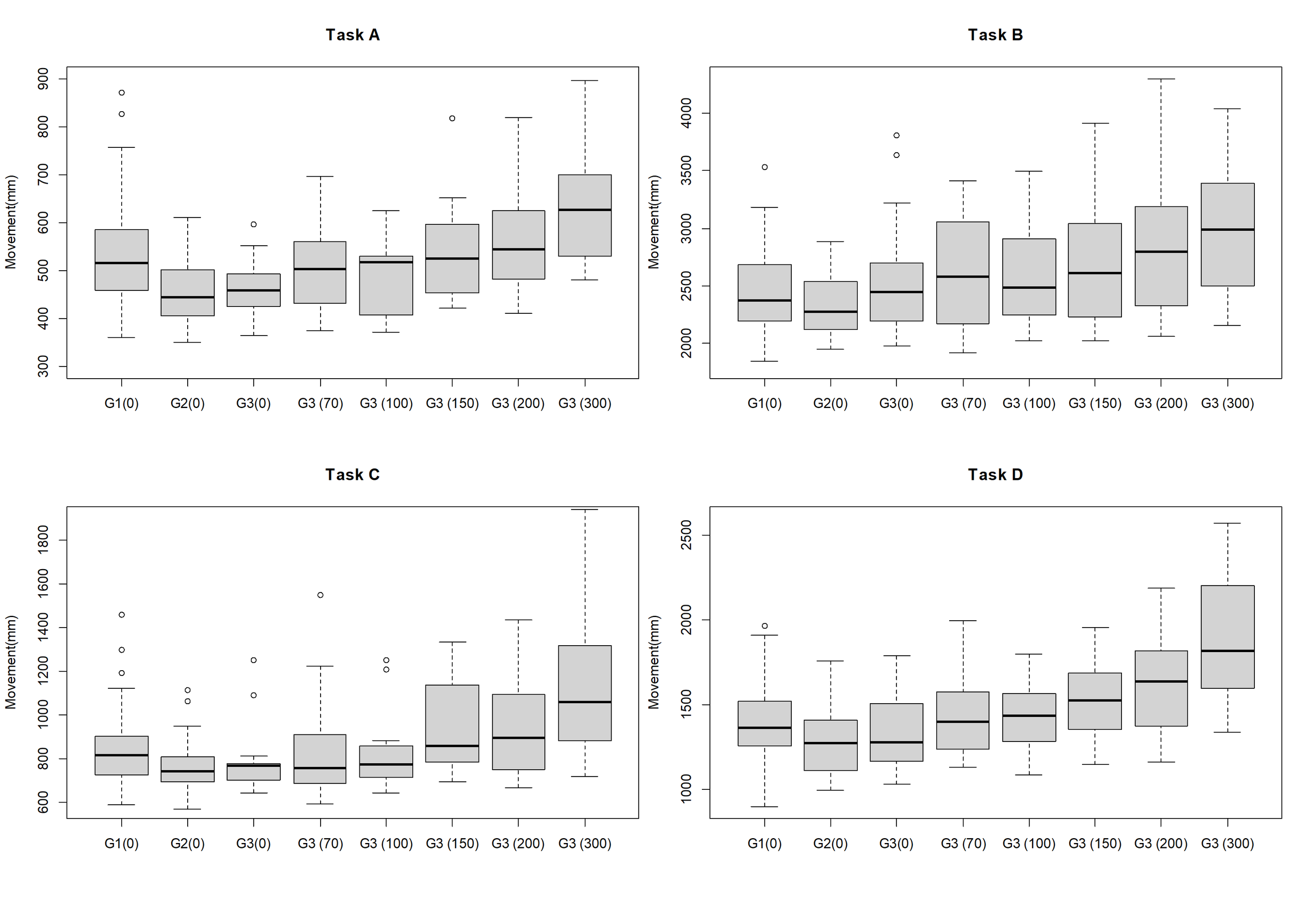

Supplement: S4 Fig — G1(0), Group 1 without delay; G2(0), Group 2 without delay; G3(0), Group 3 without delay; G3 70, Group 3 with delay of 70 ms; G3 100, Group 3 with delay of 100 ms; G3 150, Group 3 with delay of 150 ms; G3 200, Group 3 with delay of 200 ms; G3 300, Group 3 with delay of 300 ms. (TIF) [file pone.0274328.s004.tif]
